# Integrated Green Chemical Approach to the Medicinal Plant *Carpobrotus edulis* Processing

**DOI:** 10.1038/s41598-019-53817-8

**Published:** 2019-12-03

**Authors:** Sergiy Lyubchyk, Olesia Shapovalova, Olena Lygina, Maria Conceiçao Oliveira, Nurbol Appazov, Andriy Lyubchyk, Adilia Januario Charmier, Svetlana Lyubchik, Armando J. L. Pombeiro

**Affiliations:** 1 REQUIMTE, Faculdade de Ciências e Tecnologia, Universidade Nova de Lisboa, Quina de Torre, 2829-516 Caparica, Portugal; 2 CQE-Centro de Química Estrutural, Instituto Superior Tecnico, Universidade de Lisboa, Av. Rovisco Pais 1, 1049-001 Lisbon, Portugal; 3 laboratory Physical and chemical methods of analysis, Korkyt Ata Kyzylorda State University, Tulkibaeva street 7, 120008 Kyzylorda, Kazakhstan; 4 i3N/CENIMAT, Department of Materials Science, Faculty of Science and Technology, Universidade NOVA de Lisboa and CEMOP/UNINOVA, Campus de Caparica, 2829-516 Caparica, Portugal; 5 School of Engineering, Universidade Lusófona de Humanidades e Tecnologias, Campo Grande 376, 1749-024 Lisboa, Portugal

**Keywords:** Mass spectrometry, Chemical engineering, Microwave chemistry

## Abstract

Many plants have medicinal properties due to substances known as phytochemicals. To utilize these plants in practice, numerous procedures, such as extraction, isolation and characterization methods and toxicology and bioactivity studies, must be designed and implemented. Integrated approach to process *Carpobrotus edulis*, a weed medicinal plant widely spread in Portugal, was developed into a closed loop of two processes: microwave assisted extraction (MAE) and activation (MAA), to produce both phytochemicals and biochar. The use of MAE for phytochemical extraction was shown to be more energy efficient than conventional Soxhlet extraction: the process time was decreased by 7–8 times, and the energy efficiency was increased by up to 97%. The yield of the extracts is of 27%. Qualitative and quantitative identification/characterization of the phytochemicals were performed by LC-MS and phytochemical screening assays. The results clearly indicated that *Carpobrotus edulis* is rich by flavonoids (up to 24%). The use of MAA to process the residual biomass could shorten the activation time, resulting in reduced energy consumption. Biochar with a high yield of 65% (on a biomass basis) and a well-developed texture (surface area of 68.9 m^2^/g; total pore volume of 0.10 cm^3^/g; micropore volume of 0.07 cm^3^/g) is obtained.

## Introduction

For thousands of years, humans have used plant sources to fight illnesses. Initially, plants were used to brew herbal teas and to make a variety of infusions. To date, a number of different techniques have been developed for the efficient purification, separation and characterization of individual compounds and their mixtures, which have a variety of useful biologically active properties, such as antiviral, antioxidant, and antibacterial properties. The European phytochemical and plant extract market is estimated to grow from $833.7 million in 2014 to $1.25 billion by 2019, driven by the increasing health awareness of consumers in the region^1^.

According to the World Health Organization (WHO), nearly 20,000 medicinal plants exist in 91 countries^2^. Furthermore, all countries have at least one medicinal plant that grows like a weed and does not require special care.

The wild-growing plant *Carpobrotus edulis* (*C. edulis*) is a fast-growing weed with pronounced medicinal potential^3^ that flourishes along coastal areas in many parts of the world^4^, such as the Mediterranean, South Africa, North and South America, Australia, etc. *C. edulis* leaf juice, a non-destructive use of the plant, is well known for its wide range of antifungal and antibacterial external applications^5^, such as the treatment of diarrhoea, eczema, tuberculosis, throat and mouth infections; soothing itching caused by spider and tick bites; and the treatment of wounds and burns^6–8^. These effects are due to the presence of bioactive compounds—phytochemicals—in the juice. However, the phytochemical composition varies depending on the location of the natural source of the plant, causing differences in bioactivity among samples of the same species of plant from different regions^9–11^.

Quantitative phytochemical analysis of *C. edulis* extracts has revealed a high percentage of phytochemicals from the phenolic family (up to 50–60%); this family is well known and widely used because of its strong antioxidant properties and high biological activities^12^.

To utilize such biologically active compounds in practice, several procedures must be conducted, namely, extraction, isolation, characterization and/or phytochemical screening, toxicological evaluation, extended testing of *in vitro* and *in vivo* bioactivity, etc.^13^.

Another problem is that none of the currently known and used plant processing techniques meet all the requirements of economics, safety and scalability. The primary challenges in the economically viable commercial production of phytochemicals lie in the high feedstock cost, the high extraction cost of valuable bioactive compounds, their relatively low yield, and the consequently high content of residual waste biomass^14,15^.

The use of abundant weed plants (called low-value feedstocks) could help to overcome some of the mentioned limitations. However, while the technical feasibility of phytochemical extraction from weed plants has been demonstrated^16,17^, there have still been few advances in the economic feasibility of phytochemical production from weed plants due to the low yield of the target valuable products (1–15%) and the large amount of residual waste biomass (up to 99%) to be disposed^17^.

Therefore, in the present study, a widely distributed plant along the coastal zone of Portugal, *C. edulis*, was chosen as a renewable low-cost feedstock for advanced processing development. First, there is a need for additional research into the extraction of valuable phytochemicals from Portuguese sources of this weed plant. Second, based on our preliminary techno-economic estimations, it is also expected that the introduction of additional recovery pathways for the biomass waste (solid residue) obtained after extraction will help meet the requirements of economic feasibility for phytochemical extraction from weed plants^18–20^. The development of a biomass utilization pathway accompanying the extraction process will lead to an almost “zero-waste” full-chain network of valuable products (both primary (phytochemicals) and secondary (biochar)), which could be applicable to a variety of low-value feedstocks and thus aid in the creation of economically feasible weed plant processing schemes for practical usage in the future^21,22^.

Therefore, the main aim of the present work is to develop a feasible lab-scale scheme for medicinal weed plant processing. To achieve this aim, the proposed advanced *C. edulis* processing scheme is designed to be an integrated closed loop consisting of two green processes: (i) microwave-assisted extraction (MAE) to produce phytochemicals (with an emphasis on the flavonoid sub-family) and (ii) microwave-assisted activation (MAA) to produce bio-fertilizer from the residual biomass (waste after extraction).

The overall work is also designed to comply with green chemistry principles^23^, namely, with the following five: preventing waste, safer chemicals and products, safer solvents and reaction conditions, increasing energy efficiency and renewable stocks. Furthermore, the work is based on a chemical engineering approach, in which chemical processes are designed to convert raw materials into valuable products for further practical usage.

## Results and Discussion

### Quantitative characterization of the extracts

A number of solvents/solvent mixtures, such as H_2_O, MeOH, EtOH and EtOH/H_2_O with different ratio, were studied for the extraction of the phytochemicals from *C. edulis* using MAE. The conditions of the extraction were optimized to maximize the yield of the phenolic compound family, with an emphasis on the flavonoid sub-family; this yield was determined quantitatively using phytochemical screening assays, as described in experimental part 2.3.2. Data are presented in Table 1.

**Table 1:** Optimization of the conditions for MAE in terms of the yield of phenolics and flavonoids detected using phytochemical screening assays.

Conditions	Total phenolics (% w/w)	Total flavonoids (% w/w)
**SOLVENT**		
H_2_O	14.95 ± 0.51	15.06 ± 0.11
MeOH	19.83 ± 0.84	15.40 ± 0.48
EtOH	20.57 ± 0.23	15.29 ± 0.19
EtOH(70)/H_2_O(30)	22.34 ± 0.95	17.89 ± 0.32
EtOH(50)/H_2_O(50)	21.89 ± 1.72	16.62 ± 0.55
EtOH(30)/H_2_O(70)	22.30 ± 1.49	17.85 ± 0.47
**RATIO of RAW MATERIAL/SOLVENT [m/v]**		
1:05	10.29 ± 0.39	7.67 ± 0.34
1:10	14.91 ± 0.43	10.15 ± 0.16
1:15	22.30 ± 1.49	17.85 ± 0.47
**TIME of EXTRACTION [min]**		
15	22.30 ± 1.49	17.85 ± 0.47
20	22.92 ± 0.42	18.74 ± 0.38
25	23.30 ± 0.82	20.76 ± 0.78
30	23.53 ± 0.88	21.31 ± 0.32
35	24.12 ± 1.59	22.77 ± 0.65
40	24.41 ± 0.53	23.16 ± 0.46
45	24.28 ± 0.23	23.01 ± 0.99
50	24.45 ± 0.97	23.09 ± 0.41
**TEMPERATURE of EXTRACTION [°C]**		
70	24.41 ± 0.53	23.16 ± 0.46
80	24.76 ± 0.75	22.67 ± 1.02
90	23.18 ± 0.65	21.35 ± 0.37
100	23.44 ± 0.44	23.93 ± 0.94
**NUMBER of EXTRACTIONS [number of operation cycles]**		
1	24.41 ± 0.53	23.16 ± 0.46
2	9.01 ± 0.59	7.96 ± 0.37
3	3.35 ± 0.71	2.70 ± 0.35
4	1.61 ± 0.15	1.12 ± 0.34
5	0.48 ± 0.09	0.21 ± 0.14
6	0.16 ± 0.04	0.00 ± 0.00

The EtOH/H_2_O extract exhibited the highest yield of targeted phenolics (up to 21–22%) and flavonoids (16–18%). While, phytochemicals yields were similar in a case of different ratio (30%, 50% and 70%) of the EtOH/H_2_O solvent. Therefore, to address the green chemistry principle on safer solvents and auxiliaries for further optimization of MAE conditions, the solvent with maximum water content, i.e. EtOH (30%)/H2O (70%) was chosen.

The optimization conditions for MAE was carried out in a stepwise manner for ratio of raw material/solvent (1/5, 1/10, or 1/15 (m/v)); time (15, 20, 25, 30, 35, 40, 45 or 50 min); temperature (70, 80, 90, or 100 °C); and number of repeats of the extraction process (1, 2, 3, 4, 5, or 6 times).

Based on the data from the phytochemical screening assays, the following optimal conditions for the MAE process were determined: solvent - mixture of EtOH (30%) and H_2_O (70%); ratio of raw material/solvent -1/15 (m/v); extraction time −40 min; and temperature −70 °C.

Quantitative phytochemical analysis using phytochemical screening assays was also performed for the extracts obtained through Soxhlet extraction. The conditions for Soxhlet extraction were adjusted based on the optimal conditions obtained for MAE, namely, the optimal solvent [mixture of EtOH (30%)/H_2_O (70%)] and optimal ratio of material/solvent [1/15 (m/v)].

Using the same optimization procedure (i.e., based on the yield of the target phytochemicals), the following optimal conditions for Soxhlet extraction were determined (Table 2): extraction time − 260 min; and temperature −86 °C.

**Table 2:** Optimization of the conditions for Soxhlet extraction in terms of the phenolics and flavonoids detected using phytochemical screening assays.

Conditions	Total phenolics %	Total flavonoids %
**SOLVENT**		
EtOH (30)/H_2_O (70)	27.67 ± 1.10	23.61 ± 1.54
**RATIO of RAW MATERIAL/SOLVENT [m/v]**		
1:15	27.67 ± 1.10	23.61 ± 1.54
**TIME of EXTRACTION [min]**		
100	14.07 ± 0.66	13.56 ± 0.33
180	22.49 ± 1.07	20.80 ± 0.44
260	27.67 ± 1.10	23.61 ± 1.54
**TEMPERATURE of EXTRACTION [°C]**		
86	27.67 ± 1.10	23.61 ± 1.54

The optimal conditions for MAE were compared with optimal conditions for Soxhlet extraction in terms of energy efficiency (Table 3) [energy consumption required to produce the same amount of the target phytochemicals using the same optimal solvent time and initial ratio of materials]. The energy consumption was calculated according to Eq. (1), taking into account the power rating of the device (either microwave oven or Soxhlet) (in kW) multiplied by the operation time (in hours) required to produce the target phytochemicals.

**Table 3:** Comparison of the extracts obtained under optimal conditions in terms of energy consumption required to obtain the optimal yield of phenolics and flavonoids.

Extraction type	*Total phenolics, %*	*Total flavonoids, %*	T, °C	t, min	Energy consumption, kWh
Soxhlet	27.67 ± 1.10	23.61 ± 1.54	86	260	1.084
MAE	24.41 ± 0.53	23.16 ± 0.46	70	40	0.033

Comparison of the results confirmed that the energy consumption required to deliver the target phytochemicals at the desired yield by Soxhlet extraction was 32 times higher than that for the MAE process. This difference means that the energy efficiency of MAE is 97% greater than that of the Soxhlet extraction process.

### Qualitative characterization of the extracts

**Figure 1:**
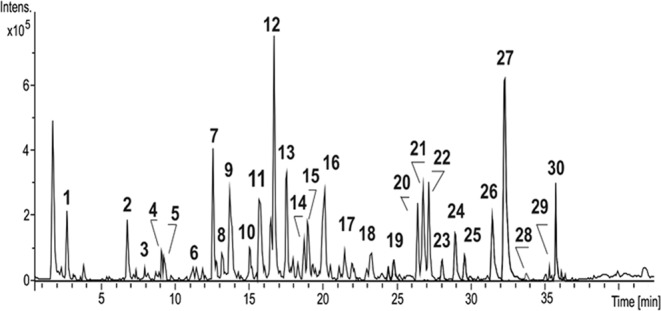
HPLC-HRMS base peak chromatogram acquired in ESI negative mode for the aqueous extract of *C. edulis* obtained by MAE. Peak numbers refer to Table 4.

The results of LC-MS analysis of the EtOH (30%)/H2O (70%) extract obtained under optimal conditions of MAE are presented in Fig. 1.

#### Composition of phenolic compounds

The *C. edulis* extract fingerprint was obtained by HPLC-DAD. The chromatographic profile indicated the separation of 30 main compounds that, based on the observed UV absorption maxima, fall into two subclasses of phenolic compounds. The main group of peaks appearing between 12 and 25 min exhibit absorbance maxima at approximately 278–280 nm, suggesting the presence of flavan-3-ols and proanthocyanidins (PAs), whereas the eleven peaks eluting between 26 and 37 min present UV-Vis absorption maxima at approximately 268 and 350 nm, revealing flavonol structures containing an *O-*glycan part in their skeleton. Figure 1 shows the base peak chromatogram obtained by LC-ESI(-)/QTOFMS analysis of *C. edulis* extract prepared by MAE. The 30 compounds were identified on the basis of their accurate *m/z* values as deprotonated molecules [M-H]^−^ and MS/MS spectra. Polyphenol identification and peak assignment were also based on comparison with similar published data. Table 4 presents the retention times, molecular formulas, exact and accurate [M-H]^−^ (*m/z*) values, and main MS^2^ product ions for each compound.

**Table 4:** HPLC-HRMS/MS identification of phenolic compounds in the EtOH(30)/H_2_O(70) extract of *C. edulis*.

Peak	Rt (min)	Proposed compound	MF	[M-H]^−^or [M-H]^2−^		Error (ppm)	MS^2^ main fragments *m/z*
				Obs. *m/z*	Cal. *m/z*		
1	2.4	Quinic acid	C_7_H_12_O_6_	191.0198	191.0197	−0.3	173.0098; 111.0090
2	5.5	Protocatechuic acid-*O*-glucoside	C_13_H_16_O_9_	315.0724	315.0711	−4.0	152.0116; 108.0226
3	8.0	Vanillic acid-glucoside	C_14_H_18_O_9_	329.0867	329.0877	−3.0	249.0613; 167.0350
4	9.1	Catechin-*O*-glucoside	C_21_H_24_O_11_	451.1247	451.1246	−0.3	289.0723; 173.9560
5	9.4	Catechin-di-*O*-glucoside	C_27_H_34_O_16_	613.1780	613.1763	−2.7	451.1250; 289.0722
6	11.6	Catechin-*O*-glucoside isomer	C_21_H_24_O_11_	451.1257	451.1246	−0.3	289.0723; 245.0821
7	12.6	B-type procyanidin dimer	C_30_H_26_O_12_	577.1358	577.1341	−2.9	407.0779; 289.0722
8	13.2	Taxifolin-glucoside	C_21_H_22_O_12_	465.1043	465.1027	−3.3	303.0482
9	13.6	(+)-catechin	C_15_H_14_O_6_	289.0745	289.0706	−1.3	—
10	15.1	B-type procyanidin trimer	C_45_H_38_O_18_	865.2011	865.1975	−4.1	695.1409; 577.1359 407.0777; 289.0722 287.0564
11	15.8	Taxifolin-glucoside isomer	C_21_H_22_O_12_	465.1050	465.1027	−5.0	303.0488
12	16.6	B-type procyanidin dimer	C_30_H_26_O_12_	577.1358	577.1341	−2.7	407.0777; 339.0879 289.0722
13	17.5	(-)-epicatechin	C_15_H_14_O_6_	289.0718	289.0706	−4.2	245.0827
14	19.0	B-type procyanidin pentamer	C_75_H_62_O_30_	720.1585	720.1579	−0.8	(289.0720 + 1152.2520)
15	19.3	B-type procyanidin hexamer	C_90_H_74_O_36_	864.1918	864.1896	−2.5	(575.1191 + 1154.2621) (289.0707 + 1440.3202)
16	20.2	B-type procyanidin trimer	C_45_H_38_O_18_	865.1998	865.1975	−2.7	695.1409; 577.1352 407.0776; 289.0719 287.0564
17	22.0	B-type procyanidin tetramer	C_60_H_50_O_24_	1153.2631	1153.2608	−2.0	983.2058; 865.1991 577.1351; 575.1204 413.0886; 287.0577
18	23.4	B-type procyanidin pentamer	C_75_H_62_O_30_	720.1591^(a)^	720.1579	−1.7	(289.0720 + 1152.2408)
19	24.8	B-type procyanidin hexamer	C_90_H_74_O_36_	864.1921^(a^)	864.1896	−2.9	(575.1191 + 1154.2652) (289.0721 + 1440.3093)
20	26.4	Isorhamnetin-3-*O*-rutinoside -rhamnoside	C_34_H_42_O_20_	769.2201	769.2197	−0.5	623.1635; 315.0574; 314.0439; 299.0208
21	26.8	Laricitrin-3-*O*-rutinose	C_28_H_32_O_17_	639.1565	639.1555	−1.6	493.0975; 373.0574 331.0448; 330.0386 315.0150
22	27.2	Syringetin-3-*O*-rutinoside-rhamnoside	C_35_H_44_O_21_	799.2306	799.2291	−1.8	635.1620; 345.0606 344.0541; 329.0603
23	28.1	Isorhamnetin-3-*O*- rutinoside- pentoside	C_33_H_40_O_20_	755.2041	755.2029	−1.6	623.1619; 315.0503 314.0436; 299.0213
24	29.1	Isorhamnetin-*O*-rutinoside	C_28_H_32_O_16_	623.1625	623.1607	−2.9	477.1000; 357.0600 315.0500; 314.0437
		Syringetin-3-*O*-rutinoside-pentoside	C_34_H_42_O_21_	785.2162	785.2146	−2.1	653.1744; 345.0615 344.0548; 329.0322
25	29.6	Syringetin-3-*O*-rutinoside	C_29_H_34_O_17_	653.1721	653.1712	−1.2	345.0605; 344.0543 329.0304
26	31.2	Isorhamnetin-*O*-rutinoside	C_28_H_32_O_16_	623.1618	623.1607	−1.7	357.0613; 315.0504 314.0432; 299.0198
27	32.2	Syringetin-*O*-rutinoside	C_29_H_34_O_17_	653.1717	653.1712	−0.7	387.0729; 345.0609 344.0539; 330.0375 329.0302
28	33.6	Syringetin-*O*-glucoside	C_23_H_24_O_13_	507.1146	507.1133	−2.6	345.0598; 344.0541 329.0302
29	35.1	Phloretin-glucoside	C_21_H_24_O_10_	435.1296	435.1286	−2.2	276.0396; 167.0347
30	35.8	Syringetin-*O*-rutinoside-glucuronyl-pentoside	C_44_H_50_O_24_	961.2637	961.2608	−3.0	799.2117; 785.2147 767.2034; 345.0611 344.0541

MF, molecular formula of the proposed compound; Rt (min), retention time in minutes; [M-H]^-^/[M-H]^2-(a)^ Obs. *m/z* and Cal. *m/z*, accurate measured mass and exact mass for the monocharged and doubly charged deprotonated molecules.

Peaks **1**, **2** and **3** (Rt = 2.4, 5.5 and 8.0 min) with deprotonated molecules at *m/z* 191.0197, 315. 0724 and 329.0867 were attributed to phenolic acid derivatives based on their accurate MS and MS^2^ data.

Peaks **8** and **11** (Rt = 13.2 and 15.8 min) displayed an ion with *m/z* 465.1047 in the negative ESI mass spectra, which produced product ions in the MS^2^ spectra with *m/z* 303.0482 due to the loss of 162 u, indicating the presence of a glucose residue linked to a taxifolin aglycone. Compounds **8** and **11** were fully identified based on their accurate mass measurements as taxifolin-*O*-glucoside isomers.

The thirteen peaks (**4**, **5**, **6**, **7**, **9**, **10**, **12**, **13**, **14**, **15, 16**, **17**, **18** and **19**) observed in the ESI (-) base peak chromatogram (Fig. 1) were identified as flavan-3-ols and PAs, a class of oligomers of catechins and their enantiomers. The compounds were tentatively assigned based on accurate mass measurement data and by comparison with published data^24–27^.

Peaks **4**, **5**, and **6** (Rt = 9.1, 9.4 and 11.6) were attributed to catechin-*O*-glycoside derivatives based on the MS^2^ spectra, which displayed an ion with *m/z* 289.0723 attributed to deprotonated catechin, resulting from the loss of one or two hexose residues. Compounds **4** and **6** were identified as catechin-*O*-glucoside isomers, whereas compound **5** was attributed to a catechin-*O*-diglucoside.

Peaks **7** and **12** (Rt = 12.6 and 16.6 min) produced deprotonated molecules with *m/z* 577.1358 in the MS spectra, which yielded peaks at *m/z* 425.0886, 407.0772 and 289.0722 as the most abundant fragment ions in the MS^2^ spectra. The loss of 152 u via a retro Diels-Alder reaction indicates a fragmentation pattern characteristic of proanthocyanidin dimers of the type (epi)catechin-(epi)catechin. Compounds **7** and **12** were assigned as B-type procyanidins.

Peaks **9** and **13** at Rt 13.6 and 17.5 min, respectively, which had deprotonated molecules with accurate *m/z* 289.0745 and 289.0718, were assigned to (+)-catechin and (−)-epicatechin.

Peaks **10** and **16** (Rt = 15.1 and 20.2 min) were attributed to B-type procyanidin trimers based on their deprotonated molecules at *m/z* 865.2011 and 865.1998, respectively, and their main fragment ions at *m/z* 577.1352, 289.0719 and 287.0564.

Peaks **14** and **18** (Rt = 19.0 and 23.4 min), yielding doubly charged species [M-H]^2−^ in their MS spectra with accurate *m/z* 720.1585 and 720.1591, respectively, were identified as b-type procyanidin pentamers. The main fragmentation pathway in the MS^2^ spectra corresponds to a pair of two monocharged species: one at *m/z* 289.0720 (base peak) assigned to a deprotonated (epi)catechin, and the other at *m/z* 1152.2520, which corresponds to a deprotonated molecule of a b-type procyanidin tetramer.

Peaks **15** and **19** (Rt = 19.3 and 24.8 min) were also attributed to doubly charged species based on the isotopic distribution observed for the deprotonated molecules [M-H]^2−^ with *m/z* 864.1918. Compounds **15** and **19** were identified by accurate mass measurements as b-type procyanidin hexamers.

Peak **17** at Rt = 22.0 min presented a signal at *m/z* 1153.2631 in the MS spectrum, which yielded main product ions with *m/z* 983.2058, 865.1919, 577.1204 and 287.0564 in the MS^2^ spectrum, suggesting a species composed of 4 (epi)catechin units. Based on the accurate data, compound **17** was identified as a b-type procyanidin tetramer.

Peaks **20**–**30** shown in Fig. 1 at higher retention times were attributed to *O*-methylated flavonol derivatives based on their accurate MS and MS^2^ fragmentation behaviour under ESI negative ion mode analysis.

Peaks **20, 21, and 22** (Rt = 26.4, 26.8 and 27.2 min) exhibited deprotonated molecules with *m/z* 769.2201, 639.1565 and 799.2306, which gave peaks at *m/z* 315.0574, 331.0448 and 345.0606 in the MS^2^ spectra, respectively, attributed to the aglycone ions (Y_0_)^−^. The first and last peaks result from the loss of 454 u (146 u + 308 u), indicating that the glycoside part contains two deoxyhexoses (146 u) and one hexose (162 u) units. Intense signals at *m/z* 314.0439, 330.0386 and 344.0541 were also observed, assigned to (Y_0_^−^H)^−•^; the presence of these radical ions indicates *O*-di-glycoside derivatives^28^. The MS^2^ spectrum also displayed signals at *m/z* 299.0208, 315.0150 and 329.0603, arising from the fragmentation of the aglycone part. The loss of 16 u suggests an *O*-methylated flavonol with methoxyl groups attached to ring B. Based on literature data^29^, compounds **20, 21 and 22** were assigned to isorhamnetin-3-*O*-rutinoside-rhamnoside, laricitrin-3-*O*-rutinose and *syringetin-3-O-rutinoside-rhamnoside, respectively*.

Peak **23** (Rt = 28.1 min) showed a deprotonated molecule at *m/z* 755.2041 and MS^2^ fragments at *m/z* 315.0500 and 314.0437, corresponding to the isorhamnetin aglycone ion (Y_0_)^−^ and its radical ion (Y_0_^−^H)^−•^, respectively. A peak at *m/z* 623.1619 (−132 u) confirmed the presence of a pentose residue on the sugar moiety. Based on the accurate mass measurements, compound **23** was attributed to isorhamnetin-3-*O*-rutinose-pentoside.

As shown in Table 4, two compounds coeluted in peak **24** at a retention time of 29.1 min. The MS spectrum displayed two peaks at *m/z* 785.2162 and 623.1625, which generated product ions at *m/z* 345.0615 and 315.0500 in the MS^2^ analysis, assigned to syringetin and isorhamnetin aglycones, respectively. The MS^2^ spectra of both precursor ions also presented characteristic abundant radical ions (Y_0_^−^H)^−•^ (*m/z* 344.0548 and 314.0436, respectively), confirming the presence of *O*-di-glycosides. Peak **24** was also attributed to two *O*-methylated flavonols, syringetin-*3-O-rutinoside-pentoside* and isorhamnetin-3-*O*-rutinoside.

The base peak chromatogram also showed peaks **25, 26, 27 and 28** (Rt = 29.6, 31.2, 32.2 and 33.6 min), yielding deprotonated molecules at *m/z* 653.1721, 623.1618, 653.1717 and 507.1146, respectively. Precursor ions at *m/z* 653.1721 and 507.1146 fragmented into syringetin aglycone at *m/z* 345.0609, whereas the precursor ion at *m/z* 623.1618 lost 308 u, leading to *m/z* 315.0504, which corresponds to isorhamnetin aglycone. Based on these accurate data, compounds **25**, **27** and **28** were assigned to syringetin-*O*-glycoside derivatives: compounds **25** and **27** were attributed to syringetin-*O*-rutinoside isomers, **26** was assigned to an isorhamnetin-*O*-rutinoside isomer, and **28** was assigned to *syringetin-O-glucoside*.

Peak **29** at Rt = 35.1 min exhibited a deprotonated molecule at *m/z* 435.1296 in the MS spectrum, which fragmented into two abundant ions at *m/z* 276.0396 (loss of 162 u) and 167.0347. Based on the accurate mass measurements, compound **29** was identified as a dihydrochalcone derivative, *phloretin-glucoside*.

Peak **30** at Rt = 35.8 min had a deprotonated molecule at *m/z* 961.2617, which presented the characteristic ion of syringetin aglycone at *m/z* 345.0611 in the MS^2^ spectrum. The MS^2^ spectrum also showed two peaks at *m/z* 799.2117 and 767.2038, corresponding to the loss of 162 and 176 u, respectively, indicating the presence of hexoses and glucuronyl moieties in its skeleton^30^. Compound **30** was identified as syringetin-rutinoside-glucuronyl-pentoside based on reported data.

The obtained results for the *C. edulis* extracts clearly indicated that this natural widely distributed weed and medicinal plant is rich in B-type procyanidin oligomers, dihydroquercetin derivatives and O-methylated flavonol derivatives. From thirty separated peaks, three correspond to the three pure compounds from phenolic acids family and other twenty-seven peaks are related to seven different flavonoids compounds known as chemopreventive agents in cancer therapy and strong inhibitors of free radical formation^31^.

### Biomass MAA. biochar properties

The main objective of this part of work was elaboration of the Microwave Assisted Activation (MAA) process to proceed with the residual biomass to address the resulted solid product -biochar.

The effect of the microwave operational parameters, including temperature (200–350 °C) and irradiation time (5–30 min), at a constant power of 600 W on the biochar yield and its textural characteristics, such as the BET surface area, total pore volume, pore size distribution, and the point of zero charge (PZC value), were investigated to optimize MAA conditions.

#### Effect of temperature

**Figure 2:**
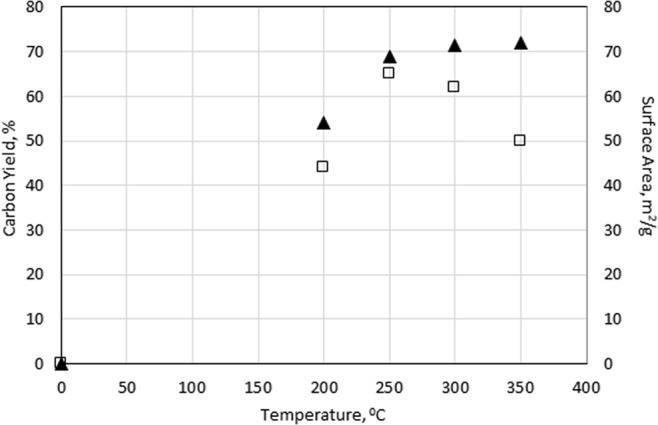
Effect of temperature on π yield and ▲ surface area of the resulting biochar at a fixed irradiation time of 10 min and constant microwave power of 600 W.

Figure 2 presents the effect of temperature on biochar yield (open squares) and specific surface area (filled triangles) after MAA biomass processing. In the temperature range deployed (200–350 °C), the yield of biochar was maximum at 250–300 °C and then decreased with further increases in temperature. The surface area of the resulting biochar product continuously increased with temperature and ranged from 54 to 72 m^2^/g under the chosen experimental conditions. At high temperatures of ≥350 °C, over gasification most likely occurs, resulting in reduced surface area/porosity with progressively decreasing carbon yield^32^.

Therefore, it can be concluded that MAA can be applied as a thermal biomass processing technique to produce a biochar product with high quality and high yield (ca. 60-65%) at a relatively low temperature (approximately 250 - 300 °C) in a short activation time (10 min), while in the case of conventional thermal biomass processing (pyrolysis or/and gasification), heating at a higher temperature and longer activation time is required to obtain similar results^32^.

#### Effect of microwave irradiation time

**Figure 3:**
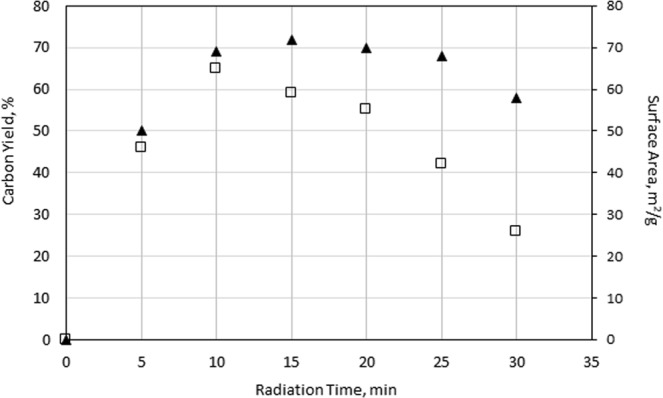
Effect of irradiation time on π yield and ▲ surface area of the resulting biochar at a fixed irradiation temperature of 250 °C and constant microwave power of 600 W.

Microwave irradiation time is another factor affecting the biochar yield and properties. The effect of irradiation time in the range of 5–30 min on the yield and surface characteristics of the bio-fertilizer was investigated at a constant microwave input power of 600 W and a temperature of 250 °C (Fig. 3).

Generally, both measured parameters (biochar yield and surface area) increased with time, exhibiting maximum values at an irradiation time of 10 min. Beyond this limit, the surface area parameters slightly decreased (from 72 to 58 m^2^/g), while a sharp decrease in biochar yield from 65 to 26% was observed as the irradiation time was prolonged from 10 to 30 min. The experimental results confirmed that microwave heating could shorten the activation time and, remarkably, produce a high-quality biochar.

The results implied that the optimal irradiation time (ca. 10–15 min) at the optimal temperature of 250 °C promoted thermal energy, which was applied in the activation process and enhanced the surface area (up to ca. 70 mg^2^/g) and internal porosity (up to 0.10 cm^3^/g) of the resulting biochar while producing a high product yield of 60–65%.

Therefore, the optimal conditions of the MAA process were evaluated to process waste biomass after phytochemical extraction from *C. edulis*. The conditions are as follows: irradiation time of 10–15 min, temperature in a range of 250–300 °C, and constant input microwave power of 600 W. Beyond these conditions, further microwave treatment might produce local overheating, which would considerably increase carbon burn-off, thus destroying the pore/surface texture and yield of the resulting biochar. At low microwave power levels, an irradiation time lower than 5 min and/or a temperature lower than 200 °C, no activation was observed; i.e., microwave irradiation had no effect on the surface area and total porosity of the product.

The detailed textural and surface characteristics of the biochar obtained under optimal MAA conditions are summarized in Table 5. The surface area was 68.9 m^2^/g; the total pore volume was 0.10 cm^3^/g, 0.07 cm^3^/g of which was the micropore volume; and the fixed carbon content was 64.9 wt %, expressed on a dry, ash-free *C. edulis* feedstock weight basis. The biochar PZC of 6.9 is neutral, reflecting the fact that the low temperature of the MAA process (approximately 250 °C) allowed partial retention of the acidic functional groups on the biochar surface, thus enabling the effective use of the resulting biochar as bio-fertilizer for both acidic and basic types of soil.

**Table 5:** Comparison of the technical and textural characteristics of the as-received biochar and biochar products from the literature.

Name	Fixed carbon, wt%	Volatile matter, wt%	Moisture content^*^, wt%	Ash mineral matter^*^, wt%
***Technical characteristics***				
Biochar/MAA/biomass stock [present study]	64.9^daf^	24.7 ^daf^	5.1	3.2
Biochar/MAA/Straw pellets^32^	43.0^daf^	—	6.7	3.9
Biochar/slow pyrolysis/wood^33^	22.5^daf^	16.8^**^	—	1.2^***^
Biochar/fast pyrolysis/rice straw^34^	15.2^daf^	—	—	10.1
**Name**	**Surface area, m**^**2**^**/g**	**Total pore volume, cm**^**3**^**/g**	**Micropore volume, cm**^**3**^**/g**	**Biochar point of zero charge, pH**_**PZC**_
***Textural characteristics***				
Biochar/MAA/biomass stock [present study]	68.9	0.10	0.07	6.9
Biochar/MAA/willow chips^32^	3.87	—	—	—
Biochar/slow pyrolysis/willow chips^32^	1.14	—	—	—
Biochar/slow pyrolysis/wood^33^	23	—	—	6.7
Biochar/fast pyrolysis/rice straw^34^	105	—	—	—
Activated carbon/MAA/corn cob^35^	507	0.32	0.26	—

Values expressed on a ^*daf*^ dry, ash-free feedstock weight basis; ^*^as-received biochar basis; ^**^dry, ash-free biochar basis; ^***^dry biochar basis.

The results are compared with biochar properties from the literature in Table 5, obtained from different plant-derived biomass sources using various thermal methods, including MAA. The properties of the bio-fertilizer product obtained by applying the MAA process to waste biomass are considerably different from those of products obtained from both slow and fast pyrolysis of biomass residues. Despite the low pyrolysis temperature, MAA preferentially generated biochar with the highest fixed carbon content (Table 5, MAA processes^32^ and [present work]), yielding 40–65 wt % biochar. The same tendency is evident for low-temperature slow pyrolysis conditions^33^, where the fixed carbon content of the resulting biochar is still high (ca. 22%^32^) in comparison with that obtained under high-temperature fast pyrolysis of biomass^34^.

The surface area and total porosity of the MAA biochar are higher than those obtained by slow pyrolysis in a similar temperature range above 250 °C, while the values are lower than those obtained with the use of fast high-temperature pyrolysis or microwave activation processes using chemical or physical activating agents to produce activated carbon^35^.

Several researchers have compared microwave pyrolysis with conventional pyrolysis and identified considerable differences between the thermal methods^32–36^, which were attributed to the activation process of biomass under microwave irradiation assisted by activation agents or fast and slow pyrolysis. However, to the best of our knowledge, only a few studies have reported microwave biochar production and direct comparisons of the properties of biochar obtained by conventional pyrolysis and MAA.

### Total mass balance

The total mass balance for the *C. edulis* processing method was estimated on a dry matter (water-free) basis with respect to the leaf mass (i.e., after pre-drying), which was considered to be 100%.

**Figure 4:**
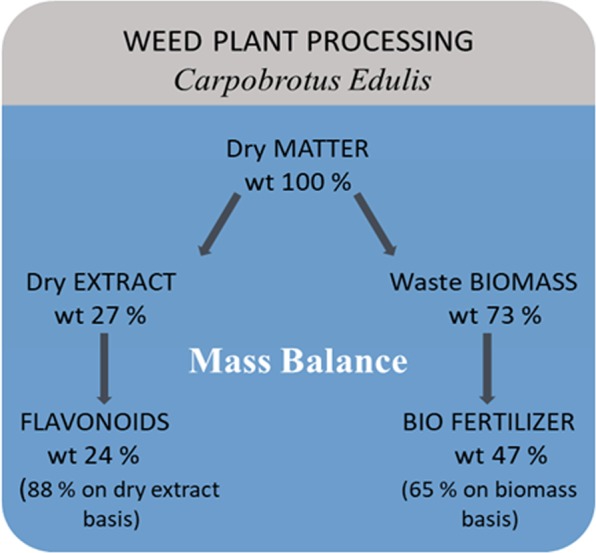
Total mass balance for the developed integrated plant material treatment process.

The total mass balance data for the *C. edulis* processing method are presented in Fig. 4 for the developed integrated process consisting of two green pathways: (i) MAE, to produce phytochemicals (with an emphasis on the flavonoid sub-family) and (ii) MAA, to produce bio-fertilizer from the residual biomass (waste after extraction).

## Conclusions

The work accomplished the design of two processes, the MAE of *C. edulis*, a natural weed plant, and MAA, to process the biomass waste after extraction, which were integrated into a single process. The result delivers two green products, phytochemicals (with a dry extract yield of 27%) and bio-fertilizer (with a yield of 47% on a dry *C. edulis* basis or 65% on a biomass weight basis). The use of MAE for phytochemical extraction from *C. edulis* was shown to be more energy efficient than conventional Soxhlet extraction: the process time was decreased by 7–8 times, and the energy efficiency was increased by up to 97%.

The results of HPLC-DAD-ESI-HRMS analysis of the aqueous extracts clearly indicated that *C. edulis* is rich in members of the flavonoid sub-family; namely, 7 of the 10 obtained pure compounds are flavonoids with strong antioxidant, anti-inflammatory, antiradical and antibacterial activities.

The experimental results showed that applying the MAA process to biomass (waste after phytochemical extraction from *C. edulis*) could shorten the activation time, resulting in reduced energy consumption, and produce a high-quality biochar. The optimal conditions for the MAA process were determined: an irradiation time of 10–15 min and temperature range from 250–300 °C at a constant input microwave power of 600 W.

Biochar with a high yield of 65% (on a biomass basis) and a well-developed texture (surface area of 68.9 m^2^/g; total pore volume of 0.10 cm^3^/g; micropore volume of 0.07 cm^3^/g; fixed carbon content of 64.9 wt %; neutral pHPZC of 6.9) was obtained. From this point of view, the obtained bio-fertilizer and its properties are comparable to analogues in the literature for both acidic and basic soil fertilization.

## Methods

### Materials

#### Plant material

*C. edulis*, a plant that is naturally widespread in Portugal, was chosen as a renewable feedstock for phytochemical production. Fresh leaves of *C. edulis* plants were collected in the coastal zone of central Portugal (38°36′58.4″N, 9°13′01.8″W). The plants were authenticated by a gardening centre (Centro de Jardinagem da Sobreda) in Setubal, Portugal. The collected material was air-dried at room temperature for 25 days, then homogenized by grinding to a fine powder and stored in airtight bottles.

#### Solvents used for the extraction step

A number of solvents and solvent mixtures were studied for the extraction of phytochemicals from *C. edulis*. In consideration of the green principle of safer solvents and noting that the application of microwave energy to highly flammable organic solvents may cause hazards, based on the literature, the following solvents were chosen as the most useful for this type of application: H_2_O, MeOH, EtOH, mixture EtOH (70%)/H_2_O (30%), EtOH (50%)/H_2_O (50%) and EtOH (30%)/H_2_O (70%).

#### Standards and reagents used for phytochemical screening assays

The chemicals and solvents used for the phytochemical screening assays were supplied by Sigma Aldrich (Steinheim, Germany) and were of analytical grade (A.R.) and/or reagent grade (R.G.). The materials included a mixture of phosphomolybdate and phosphotungstate (Folin-Ciocalteu phenol reagent); gallic acid (C_6_H_2_(OH)_3_COOH); quercetin **(**3,3′,4′,5,6-pentahydroxyflavone); rutin trihydrate (C_27_H_30_O_16_·3H_2_O, quercetin-3-rutinoside trihydrate); Na_2_CO_3_; Al_2_O_3_; CH_3_COONa; NaNO_2_; AlCl_3_ and 1 N NaOH.

The materials used for the analysis of phytochemicals in extracts, HPLC-grade methanol (99.9%), acetonitrile (99.9%) and water solution containing 0.1% (v/v) formic acid, were supplied by Sigma-Aldrich (Steinheim, Germany).

### Experimental procedures

#### Microwave-assisted extraction (MAE)

MAE was carried out using a microwave synthesis reactor (Monowave 300, Anton Paar GmbH, Austria). For the extraction, a dry crushed sample was placed in a G30 borosilicate glass vial and placed in an oven. To avoid overheating of the sample, internal temperature control was used. Namely, the “heating over time” heating mode was chosen, where the heating time from room temperature to the required temperature in the range of 70–100 °C was fixed at 3.5 min. The best solvent type for MAE was evaluated on yield of phenolics and flavonoids detected quantitatively in given extracts using phytochemical screening assays. The optimization conditions for MAE were carried out for the best solvent type in a stepwise manner for the ratio of raw material/solvent (1/5, 1/10, or 1/15 (m/v)); time (15, 20, 25, 30, 35, 40, 45 or 50 min); temperature (70, 80, 90, or 100 °C); and number of repeats of the extraction process (1, 2, 3, 4, 5, or 6 times). All MAE runs were performed at a constant power of 50 W. The optimized conditions resulting in the highest extract yield using MAE were compared with the well-known and widely used conventional method of Soxhlet extraction in terms of energy efficiency.

#### Soxhlet extraction

Soxhlet extraction was performed using a Soxhlet apparatus (Sigma Aldrich, Steinheim, Germany) with an extractor capacity of 200 ml, a flask capacity of 300 ml, and a maximum operating power of 250 W. For the extraction, 10 g of dry crushed sample was placed into the extractor in cellulose extraction thimbles. Optimization of the conditions for the Soxhlet extraction was performed considering the number of operation cycles (thus, different operation times and energy consumption values) required to obtain the same degree of product (extract) yield, while the solvent type and ratio of raw material/solvent were the same as those under the optimized conditions for MAE.

The results were compared with the MAE technique in terms of the energy consumption required to produce the same amount of extract using the same optimal type of solvent reagent and initial ratio of materials.

#### Microwave-assisted activation (MAA)

MAA without the usage of any of the conventional activation agents (chemicals, CO_2_ or steam gases) in the atmosphere of the exhaust gas was used to process the biomass remaining after extraction with the aim of producing biochar—a valuable secondary product. After pre-drying at 110 ± 5 °C for 24 h, the biomass samples (solid residue after extraction) were subjected to MAA using a modified lab-scale cavity-type microwave oven (Milestone, Italy) with continuous output power and an operating microwave frequency of 2.45 GHz (wavelength 12.2 cm). The MAA process was carried out under a constant power of 600 W. The effect of operational parameters, namely, temperature (200–350 °C) and irradiation time (5–30 min), on the biochar yield and physical characteristics was investigated. Samples (100 g of pre-dried biomass) were placed in a ceramic crucible into the microwave oven cavity and heated at an average rate of 10 °C/min until the selected temperature of carbonization (200, 250, 300 or 350 °C) was achieved. Then, the temperature was held for different times (5–30 min). Finally, the carbonized biomass (biochar) was cooled to room temperature under N_2_ flow and kept in a desiccator until physical characterization. The yield was calculated as the ratio of the dry weight of the resultant biochar to the weight of the raw pre-dried biomass precursor. The resulting biochar characteristics were compared with the available values from the literature.

### Analytical methods

#### LC–MS analysis

The EtOH(30)/H_2_O(70) *of C. edulis* obtained by MAE were analysed by high-performance liquid chromatography coupled to a diode array detector (HPLC-DAD) using an Ultimate 3000 SD (Thermo Scientific) and by HPLC coupled to high-resolution mass spectrometry (HPLC-HRMS) on an Ultimate 3000 RSL Cnano system (Thermo Scientific) interfaced with a quadrupole time-of-flight (QTOF) Impact II mass spectrometer equipped with an electrospray source (Bruker, Daltoniks). Chromatography separation was carried out on a Kinetics 5 μm C18 100 Å column (150 mm × 4.60 mm) or on a Kinetics 1.7 µm C18 100 Å LC column (150 × 2.1 mm) (Phenomenex, USA) at a constant temperature of 35 °C and flow rates of 0.300 mL min^−1^ and 0.150 mL min^−1^, respectively. The mobile phase consisted of 0.1% (v/v) formic acid in water (A) and 0.1% (v/v) formic acid in acetonitrile (B). The elution conditions were as follows: 5% B for 2 min; 5 to 30% B for 30 min; 30 to 100% B for 13 min; 100% B for 3 min; 100 to 5% 2 min; and finally, 5% B for 10 min.

The mass spectrometer was operated in ESI negative ion mode at high resolution. The optimized parameters were as follows: ion spray voltage, 2.5 kV; end plate offset, -500 V; nebulizer gas (N_2_), 2.8 bar; dry gas (N_2_), 8 L min^−1^; dry heater, 200 °C. Internal calibration was performed in high-precision calibration (HPC) mode with a solution of 10 mM sodium formate, which was introduced to the ion source via a 20 µL loop at the beginning of each analysis using a six-port valve. Sample analysis was performed by data-dependent acquisition (auto MSMS mode) in a *m/z* range of 50–1500 with a rate of 3 Hz and by a dynamic method with a fixed cycle time of 3 s. Data were processed using Data Analysis 4.1 software (Bruker Daltonics).

#### Identification of flavonoid glycosides. detection of fragmentation pathways

**Figure 5:**
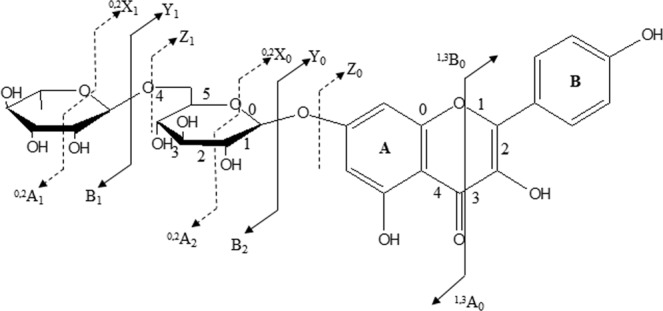
Identification of the flavonoid ions and ions created due to glycosidic bond cleavages (adapted from^37^).

To denote the various fragment ions obtained by LC-MS analysis of the *C. edulis* extract, the nomenclature for flavonoid fragmentations proposed by Claeys and that for carbohydrate fragmentations proposed by Domon and Costello^37^ and subsequently expanded by Li and Claeys^38^ were adopted and used (Fig. 5). The labels ^*i,J*^A^−^ and ^*i,J*^B^−^ have been applied to define the primary fragment ions containing intact A and B rings, respectively, and the superscripts *i* and *J* indicate the number of broken C-ring bonds. The labels Y_n_^−^and ^*i,J*^X_n_^−^ denote glycoside flavonoids. The rupture of glycosidic bonds accompanied by hydrogen transfer gives rise to ions of Y_n_^−^ type, whereas ^*i,J*^X_n_^−^ indicates ions formed by multiple cleavages involving the *i,J* bonds of carbohydrate units, where the subscript *n* indicates the number of interglycosidic bonds from aglycone.

#### Phytochemical screening assays

Phytochemical screening assays were performed to detect the total phenolic compound and flavonoid sub-family contents in the extract samples. A UV-Vis GBC 918 spectrometer was used to detect the target phytochemicals.

#### Total phenol determination

The total phenolic compounds were determined using Folin-Ciocalteu reagent^39^. Plant extract (100 μl, 0.01%), Folin-Ciocalteu reagent (500 μl) and sodium carbonate (2 ml, 2%) were thoroughly mixed and kept at room temperature for 30 min before measurements. The total phenolic concentration was determined by the absorbance at 720 nm using gallic acid as a standard at 10 to 200 μg/ml. *y* = 0.1572 *x -* 0.147, with R^2^ = 0.988.

#### Total flavonoid determination

Flavonoids were determined using the aluminium chloride colorimetric method^40^. A total of 250 μl of plant extract (0.01%) was mixed with 75 μl of NaNO_2_, 150 μl of AlCl_3_ (10%), 500 μl of NaOH (1 N) and 2.5 ml of distilled water. The mixture was held at room temperature for 5 min, after which the absorbance of the mixture was measured at 510 nm. The calibration curve was prepared using quercetin solutions at 10 to 120 μg/ml. y = 0.2832 × – 0.263, with R^2^ = 0.986.

#### Surface and texture characterization

The biochar was characterized by elemental and proximate analyses using an automatic CHNS-O elemental analyser and a Flash EATM 1112.

The porosity and surface parameters of the biochar samples were measured by means of low-temperature nitrogen adsorption isotherms at 77 K collected using a BET accelerated surface area and porosimetry analyser (Micromeritics ASAP 2010). Prior to adsorption testing, the samples were outgassed at 240 °C for 24 h under a pressure of 10^−3^ Pa.

The specific surface area was calculated using the BET equation^41^. The Dubinin-Radushkevich^42^ and Barrett-Joyner-Halenda (BJH)^43^ methods were applied to determine the micro- and mesopore volumes, respectively. The biochar point of zero charge (PZC) was obtained by acid–base titration^44^.

### Energy consumption calculation

The energy consumption (*E*) in kilowatt-hours (kWh) for a single extraction run is equal to the power *P* in watts (W) multiplied by the number of hours of extraction device usage (*t*_*h*_) per extraction process and divided by 1000 watts per kilowatt, according to Eq. (1):

1 $${E}_{(kWh)}={P}_{(W)}\times {t}_{(h)}/{1000}_{(W/kW)}$$

### Total mass balance evaluation

The total mass balance for the integrated processing scheme was estimated *via* calculation of the yield of the primary valuable product—phytochemicals, from the extraction process—and the secondary valuable product—biochar, from biomass processing. Emphasis was also given to the estimation of the yield and mass balance of the flavonoid sub-family of phytochemical products extracted from *C. edulis*. The exact conservation law was used in the analysis of the system^45^.

The moisture content of the *C. edulis* leaf mass was calculated by measuring the mass while “wet” (fresh leaves) and that after drying and using Eq. (2):

2 $$ \% Moisture=100\frac{{m}_{wet}-{m}_{dry}}{{m}_{wet}}$$

however, further calculations were performed on a dry matter (water-free) basis, i.e., with respect to the *C Edulis* leaf mass after pre-drying, which was considered to be 100%.

## Data Availability

The datasets generated during and/or analysed during the current study are available from the corresponding author on reasonable request.

## References

[1] Research and Markets. Europe phytochemicals and plant extracts market by applications - analysis and forecast to 2019. http://www.researchandmarkets.com/research/x6lww9/europe (2015).

[2] World Health Organisation (WHO), World Conservation Union (IUCN) & World Wide Fund for Nature (WWF). *Guidelines on the Conservation of Medicinal Plants*. (WHO/IUCN/WWF, 1993).

[3] Vilà M (2008). Widespread resistance of Mediterranean island ecosystems to the establishment of three alien species. Divers. Distrib..

[4] Schierenbeck KA, Symonds VV, Gallagher KG, Bell J (2005). Genetic variation and phylogeographic analyses of two species of *Carpobrotus* and their hybrids in California. Mol. Ecol..

[5] Tamilselvan N, Thirumalai T, Shyamala P, David E (2014). A review on some poisonous plants and their medicinal values. Journal of Acute Disease.

[6] Martins A (2011). Antibacterial properties of compounds isolated from *Carpobrotus edulis*. Int. J. Antimicrob. Agents.

[7] van der Watt E, Pretorius JC (2001). Purification and identification of active antibacterial components in *Carpobrotus edulis* L. J. Ethnopharmacol..

[8] Martins M (2005). Inhibition of the *Carpobrotus edulis* methanol extract on the growth of phagocytosed multidrug-resistant *Mycobacterium tuberculosis* and methicillin-resistant *Staphylococcus aureus*. Fitoterapia.

[9] Xu, Z. & Howard, L. R. *Analysis of antioxidant-rich phytochemicals* (John Wiley & Sons, 2012).

[10] Wolfender JL, Eugster PJ, Bohni N, Cuendet M (2011). Advanced methods for natural product drug discovery in the field of nutraceuticals. Chimia (Aarau).

[11] Boyer J, Liu RH (2004). Apple phytochemicals and their health benefits. Nutr. J..

[12] Omoruyi BE, Bradley G, Afolayan AJ (2012). Antioxidant and phytochemical properties of *Carpobrotus edulis* (L.) bolus leaf used for the management of common infections in HIV/AIDS patients in Eastern Cape Province. BMC Complement. Altern. Med..

[13] Sasidharan S, Chen Y, Saravanan D, Sundram KM, Latha LY (2011). Extraction, isolation and characterization of bioactive compounds from plants’ extracts. Afr. J. Tradit. Complement. Altern. Med..

[14] Gang, D. R. *Phytochemicals, plant growth, and the environment* (Springer Science & Business Media, 2012).

[15] Balandrin MF, Klocke JA, Wurtele ES, Bollinger WH (1985). Natural plant chemicals: sources of industrial and medicinal materials. Science.

[16] Azmir J (2013). Techniques for extraction of bioactive compounds from plant materials: a review. J. Food. Eng..

[17] Azwanida N (2015). A review on the extraction methods use in medicinal plants, principle, strength and limitation. Med. Aromat. Plants.

[18] Moncada J, Tamayo JA, Cardona CA (2016). Techno-economic and environmental assessment of essential oil extraction from Oregano (*Origanum vulgare*) and Rosemary (*Rosmarinus officinalis*) in Colombia. J. Clean. Prod..

[19] Harker KN, O’Donovan JT (2013). Recent weed control, weed management, and integrated weed management. Weed Technol..

[20] Koay GFL, Chuah T-G, Choong TSY (2013). Economic feasibility assessment of one and two stages dry fractionation of palm kernel oil. Ind. Crops Prod..

[21] Strezov, V. & Evans, T. J. *Biomass processing technologies* (CRC Press, 2014).

[22] Kan T, Strezov V, Evans TJ (2016). Lignocellulosic biomass pyrolysis: a review of product properties and effects of pyrolysis parameters. Renewable and Sustainable Energy Reviews.

[23] Anastas, P. T. & Warner, J. C. *Green chemistry: theory and practice* (Oxford University Press, 1998).

[24] Rothwell JA (2015). Phenol-Explorer 3.6: a major update of the Phenol-Explorer database to incorporate data on the effects of food processing on polyphenol content. Database.

[25] Lin LZ, Sun J, Chen P, Monagas MJ, Harnly JM (2014). UHPLC-PDA-ESI/HRMS^n^ profiling method to identify and quantify oligomeric proanthocyanidins in plant products. J. Agric. Food Chem..

[26] Sun B, Leandro MC, de Freitas V, Spranger MI (2006). Fractionation of red wine polyphenols by solid-phase extraction and liquid chromatography. J. Chromatogr. A.

[27] Rockenbach II (2012). Characterization of flavan-3-ols in seeds of grape pomace by CE, HPLC-DAD-MSn and LC-ESI-FTICR-MS. Food Res. Int..

[28] Vukics V, Guttman A (2010). Structural characterization of flavonoid glycosides by multi-stage mass spectrometry. Mass Spectrom. Rev..

[29] Cai W, Gu X, TaNG J (2010). Extraction, purification, and characterisation of the flavonoids from Opuntia milpa alta skin. Czech J. Food Sci..

[30] Fu F, Wang HL (2015). Metabolomics reveals consistency of the shoot system in Medicago truncatula by HPLC-UV-ESI-MS/MS. Int. J. Food Sci. Technol..

[31] Menaa, F. & Tréton, C. H. Polyphenols in chronic diseases and their mechanisms of action in *Polyphenols in human health and disease* (eds Watson, R. R., Preedy, V. R., & Zibadi, S.) 819-830 (Elsevier/Academic Press, 2014).

[32] Mašek O (2013). Microwave and slow pyrolysis biochar—Comparison of physical and functional properties. J. Anal. Appl. Pyrolysis.

[33] Ronsse F, van Hecke S, Dickinson D, Prins W (2013). Production and characterization of slow pyrolysis biochar: influence of feedstock type and pyrolysis conditions. Glob. Change Biol. Bioenergy.

[34] Wannapeera J, Worasuwannarak N, Pipatmanomai S (2008). Product yields and characteristics of rice husk, rice straw and corncob during fast pyrolysis in a drop-tube/fixed-bed reactor. Songklanakarin Journal of Science & Technology.

[35] Elsayed M, Zalat O (2015). Factor affecting microwave assisted preparation of activated carbon from local raw materials. International Letters of Chemistry, Physics and Astronomy.

[36] Brown TR, Wright MM, Brown RC (2011). Estimating profitability of two biochar production scenarios: slow pyrolysis vs fast pyrolysis. Biofuel. Bioprod. Biorefin..

[37] Domon B, Costello CE (1988). A systematic nomenclature for carbohydrate fragmentations in FAB-MS/MS spectra of glycoconjugates. Glycoconj. J..

[38] Li QM, Claeys M (1994). Characterization and differentiation of diglycosyl flavonoids by positive ion fast atom bombardment and tandem mass spectrometry. Biol Mass Spectrom.

[39] Madaan R, Bansal G, Kumar S, Sharma A (2011). Estimation of Total Phenols and Flavonoids in Extracts of Actaea spicata Roots and Antioxidant Activity Studies. Indian J. Pharm. Sci..

[40] Chang C-C, Yang M-H, Wen H-M, Chern J-C (2002). Estimation of total flavonoid content in propolis by two complementary colorimetric methods. J. Food Drug Anal..

[41] Brunauer S, Emmett PH, Teller E (1938). Adsorption of gases in multimolecular layers. J. Am. Chem. Soc..

[42] Dubinin MM, Radushkevich LV (1947). Equation of the characteristic curve of activated charcoal. Proc. Acad. Sci. USSR Phys. Chem. Sect..

[43] Barrett EP, Joyner LG, Halenda PP (1951). The determination of pore volume and area distributions in porous substances. I. Computations from nitrogen isotherms. J. Am. Chem. Soc..

[44] Sontheimer, H., Crittenden, J. C. & Summers, R. S. *Activated carbon for water treatment*. (DVGW-Forschungsstelle, 1988).

[45] http://www.ico.org/proChects/Good-Hygiene-Practices/cnt/cnt_sp/sec_3/docs_3.2/Determine%20m%20c.pdf.

